# Research use at the Ministry of Health in Burkina Faso: the decision-makers’ perspective

**DOI:** 10.1186/s43058-021-00126-9

**Published:** 2021-02-17

**Authors:** Christian Dagenais

**Affiliations:** Department of Psychology, University of Montreal, P.O. Box 6128, Centre-Ville Station, Pavillon Marie-Victorin, Bureau C-355, Montreal, QC H3C 3J7 Canada

**Keywords:** Policy-making, Decision-making, Research use, Evidence, Knowledge transfer, Qualitative, Burkina Faso, Ministry of Health

## Abstract

**Background:**

Despite the increased emphasis placed on the use of evidence for policy development, relatively few initiatives have been developed to support evidence-informed decision-making, especially in West Africa. Moreover, studies examining the conditions under which policy-makers use research-based evidence are still scarce, but they show that their attitudes and opinions about research are one of the main determinants of such use. In February 2017, Burkina Faso’s Minister of Health planned to create a unit to promote evidence-informed decision-making within the ministry. Before the unit was set up, documenting the attitudes towards research at the highest levels of his Ministry appeared profitable to the unit’s planning.

**Method:**

Individual interviews were conducted by the author with 14 actors positioned to consider evidence during decision-making from the Burkina Faso’s Minister of health cabinet. An interview grid was used to explore several themes such as attitudes towards research, obstacles and facilitators to research use, example of research use in decision-making and finally, ways to increase decision-makers’ participation in knowledge transfer activities. Interviews were partially transcribed and analysed by the author.

**Results:**

The results show a mixed attitude towards research and relatively little indication of research use reported by respondents. Important obstacles were identified: evidence inaccessibility, lack of implementation guidelines, absence of clear communication strategy and studies’ lack of relevance for decision-making. Many suggestions were proposed such as raising awareness, improving access and research communication and prioritizing interactions with researchers. Respondents agree with the low participation of decision-makers in knowledge transfer activities: more leadership from the senior officials was suggested and greater awareness of the importance of their presence.

**Conclusions:**

The conclusion presents avenues for reflection and action to increase the potential impact of the knowledge transfer unit planned within the Ministry of Health of Burkina Faso. This innovative initiative will be impactful if the obstacles identified in this study and policy-makers’ preferences and needs are taken into account during its development and implementation.

Contributions to the literature ➔ The relatively low level of research use in decision-making within Burkina Faso’s Ministry of Health is confirmed.

➔ The major obstacles identified by decision makers (e.g. attitudes towards research-based evidence, difficulty in accessing evidence and lack of implementation guidelines) should be adequately addressed to improve the situation.

➔ This study offers a pragmatic discussion on how to respond to real-world obstacles to evidence-informed policy making, and that requires a combination of interventions.

## Background

Since the emergence of the evidence-based medicine movement in the 1990s, the trend towards evidence-based practice and decision-making has grown dramatically and more and more voices are heard in favour of greater use of research by practitioners and decision-makers [1, 2]. Nevertheless, despite an increasing mobilisation of researchers and research-funding agencies, there is a persistent gap between available scientific knowledge and its use [3–6]. The scientific literature on knowledge transfer (KT) still provides little evidence on the processes that lead to the use of scientific knowledge in a given context and on its effectiveness, [7–11]. In fact, KT strategies present the characteristics of complex social interventions: they are based on several theories (organisational change, collaboration, individual behaviour, etc.), involve many actors (researchers, decision-makers and professionals from practice environments) and they imply the commitment and participation of these actors and their organisations [12]. Thus, more studies are needed to understand how research could play a role in policy and practice, especially in West Africa, where still few initiatives to support evidence-informed policy and practice are implemented and evaluated.

In February 2017, Burkina Faso’s Minister of Health, Nicolas Méda, himself a researcher, planned to create a unit to promote the use of research for decision-making within his ministry and mandated the author to support its implementation. On September 27, 2017, the Government of Burkina Faso [13] approved by decree the Ministry reorganisation proposed by the Minister and the creation of a Knowledge Management and Transfer Unit (KMTU) directly attached to his cabinet. This unit explicitly aims to develop better integration of research results into decision-making and health system management at all levels. Thus, this unit is called upon to play a knowledge brokerage role. Its mission is to inform, with evidence-based information available and in a complex environment, all public decisions on health.

This article presents the results of a study conducted after several years of research in Burkina Faso. Throughout these years, the research teams involved with the author have made considerable efforts to promote the use of research, particularly by implementing knowledge brokering activities [14–16]. These efforts have often had positive impacts at the local and regional levels, but there is little indication of any results use within the Ministry [14, 17], as it has always been difficult to involve senior officials in our research dissemination activities, or even to obtain a few minutes of conversation with them. We are only now beginning to identify the conditions under which policy-makers use research, but we know their attitudes and opinions about research are one of the main determinants of such use [18–21]. Before the KMTU was set up, the author therefore proposed to the Minister to document the attitudes towards research at the highest levels of his ministry, to better understand the potential obstacles to its implementation.

## Method

Individual interviews were conducted by the author between May 23 and 27, 2017, with 12 directors and two senior advisors (*N* = 14) of the Ministry of Health. All the directors who were in the capital during that week have been invited by telephone to participate. Nobody refused. These interviews took place in the offices of the Ministry where nobody else than the interviewee and the author was present. They lasted an average of about 45 min and were recorded digitally with the explicit agreement of the respondents, to whom anonymity was guaranteed. The interviews were intended to gather their views on the use of research for decision-making. The interview grid included five themes: (1) their perception of the general attitude towards research, (2) obstacles, and (3) elements that could foster greater research use in their department. Respondents were also asked to (4) give concrete examples of research evidence that had been used in their department and (5) explore ways to improve decision-makers’ participation in knowledge transfer activities. Interviews were partially transcribed the same day the interviews took place by the author who conducted a thematic content analysis on all of the data from the interviews [22]. The aim of the analysis was to identify the main recurring themes, but also to highlight different views and perspectives when appropriate.

## Results

### A mixed attitude towards research

While several affirmed that research was generally well accepted and that it was necessary for planning, others considered it the poor relation of the system. The former said research use was encouraged, and some even claimed it was a priority for all Ministry of Health officials. As evidence of this, they pointed to the three research centres under the Ministry’s charge.

However, a majority of respondents considered that research had very little influence on decisions, except perhaps in certain departments, such as the Directorate General for Health and the Department of Sectoral Studies and Statistics. With respect to other decision-makers, the respondents perceived indifference to some extent, and it was reported that sometimes certain people were even hostile towards research. Several respondents saw research as a world apart, an “exclusive domain” that was not a priority for them. According to one, there was a tendency to rely much more on recommendations from major NGOs and WHO than on primary research, except when there was an emergency, such as a dengue outbreak in 2016. However, some explained that the situation was changing, thanks in part to the last two ministers, both of whom had research backgrounds.

### Key obstacles to research use

Aside from the sometimes half-hearted openness to research, the two main obstacles identified by respondents were the difficulties of accessing research results and the fact that action plans to apply those results were rarely proposed. Given that “we can’t use what we don’t know exists,” the issue of access to research results is fundamental, and such access must be timely. However, the majority of respondents reported having difficulty identifying studies that could help them make decisions. This was, according to some, partly due to the fact that researchers often are not concerned about communicating their results in any way other than through scientific publications: “...the researcher does this to earn his stripes; they’ll disseminate [results] elsewhere, but not here....” Even though respondents knew a significant number of studies were being conducted, there was no registry they could consult to identify those that might be useful to them in their circumstances. They highlighted the absence of any clear communication strategy for research. The other main obstacle to use was related to researchers and the fact that they often do not make recommendations regarding how to apply the results, which reduces the likelihood that the studies conducted will lead to changes. Since “in [training] schools there is no attention given to use”, support is often needed to implement changes that could result from a study.

Another major obstacle to use associated with researchers had to do with their mode of communication, which too often uses specialised jargon that is difficult to grasp. Respondents also questioned the relevance of available studies that are not always in line with the realities confronting the Ministry: “...research is conducted that is useful to the researcher, but not necessarily to the department.” They also highlighted the difficulties of funding not only the research itself, but also its implementation.

### Several ideas to promote research use

Most respondents proposed solutions to circumvent the obstacle of access to research results. The main solution involved the coordination of results dissemination and a potential role for the Department of Archives and Documentation, which, even though its specific mandate is not well known, could set up a registry of available studies. Some respondents felt this department should be responsible for communicating results: “...they should take the lead in communicating results. If this department assumes this authority, it will certainly find the way.” Research results should be published on the Ministry’s website: “First we need to know the study exists, and then [we can] access it...” Social networks should be used to disseminate the most relevant results in the form of one-page summaries and bulletins. Results dissemination sessions need to be organised that specifically target the technical team likely to use the results. However, for decision-makers to want to access research results, they need to be told about the utility of research, shown that it can make a positive contribution, and convinced that it is in their interest to use its results: “It would take a kind of research lobbying; you have to be able to explain the interest or impact this study could have or [how it] could be used.” This could induce a “thirst for information”, and people need to develop the skills to pursue that information.

Another component of proposed solutions pertains to researchers and in particular those in centres under the charge of the Ministry of Health: “The centres need to be reminded they’re part of the Ministry; they go disseminate [their results] elsewhere, but not here... The centres should focus on themes that are priorities for the Ministry.” Several respondents stressed the importance of developing researchers’ capacities to better communicate their results. Also, researchers should not expect non-researchers to come to them, but rather should be proactive, “They know which people can use [the results].” Some respondents felt it was the researcher’s responsibility to demonstrate how implementation could have a positive impact on decisions. They underscored the advantages of involving decision-makers in research to facilitate results use, as well as the importance of senior Ministry officials being at the forefront of communications.

Finally, it was suggested that a sharing platform be set up to create an interactive process between researchers and decision-makers: “... those making policy need to see that research is important and researchers need to see that it’s important to share... to supply [information] to those who can use it.” Some thought researchers and decision-makers should meet regularly and that existing mechanisms, such as the Ministerial Sector Council (*CA du secteur ministériel* – CASEM) or the Sectoral Dialogue Forum (*Cadre sectoriel de dialogue* - CSD), could be used to invite researchers to present their work.

### Relatively little indication of research use reported by respondents

Nearly half of the respondents were unable to cite a study that had been used to make a decision or guide actions in their department. When they did, most often they referenced population or epidemiological data being used, for example, to identify districts that performed less well than others. Some mentioned recently adopted policies but were unable to cite a single study that had influenced their formulation.

However, some respondents, even if not many, were very familiar with the studies produced on free healthcare, especially those conducted in their country about two pilot projects, and were able to name the results that had been taken into account in the policy implemented. Those studies provided a clear understanding of the benefits of free healthcare and guided actions to improve maternal and child health. Other recent studies on dengue fever served to make known the presence of this disease in Burkina Faso and to raise public awareness. However, no one seemed to know how far this information had circulated. Respondents mentioned other studies, such as one about a malaria vaccine. Most often, they referred only to one study, in which they themselves had participated. Also mentioned was a study that clarified what needed to be put in place (for staff retention in rural areas), but the resources to do so did not follow.

### Encourage decision-makers’ involvement in knowledge transfer activities

At the end of the interview, I told respondents about the difficulties encountered in recent years in getting decision-makers to attend knowledge transfer activities such as dissemination workshops or deliberative dialogue sessions. Despite the author’s and his teams repeated efforts, most often the invited decision-maker either delegated a colleague or subordinate to take his place or introduced himself at the beginning of the activity and left immediately after his opening speech. For the respondents, this situation was a fact of life at the Ministry; some called it “the African affliction”—the authorities are all very busy. As a result, they tend to delegate what they know the least and give priority to what is already familiar. Their awareness needs to be raised so they will prioritise this activity, by extending the invitation in person and meeting with them to explain why they should come. Half of the respondents suggested applying the weight of the hierarchy, noting the instructions of the most senior officials: “...if it comes from the Minister or the Secretary General it will work; the man at the top needs to send a clear message.”

## Discussion

This study shows that policy-makers interviewed had mixed attitude towards research and reported very few examples of how research-based evidence informed decisions. They also identified several important obstacles: inaccessibility, lack of implementation guidelines, absence of clear communication strategy and relevance of health research studies. Many suggestions to improve research use were also proposed such as raising awareness, improving access and research communication and prioritising interactions with researchers. Finally, they agree with the low participation of decision-makers in knowledge transfer activities: more leadership from the senior officials was suggested to increase their involvement and greater awareness of the importance of their presence.

To our knowledge, the Knowledge Management and Transfer Unit (KMTU) innovative initiative is unique. Some experiments were implemented in different countries in West Africa. For example, a rapid response team was created by the African Population and Health Research Center in seven countries of West-Africa: Kenya, Rwanda, Zambia, Malawi, Sierra Leone, Burkina Faso and Liberia. The mandate of these teams was to facilitate the provision, in a timely manner, of rapidly produced, high-quality, synthesised evidence. Other initiatives of this type were integrated in a platform aims to provide quick access for policy-makers in Burkina Faso to high-quality research evidence about health systems [23]. But none of these initiatives were directly attached to the Minister’s cabinet with a clear mandate to inform all public decisions on health.

A major synthesis published in 2016 by the Alliance for Useful Evidence focused on the effectiveness of six different mechanisms aimed at increasing the use of research by decision-makers [8, 11]. This synthesis presents the results of 23 systematic reviews in the field of KT, supplemented by a scoping review in the broader social science literature. At the end of 2019, a collaborator conducted an update of the more recently published systematic reviews about these mechanisms. No results contradict Langer’s conclusions [11], and many confirm them. The six KT mechanisms identified (Table 1) as effective or promising in this review of reviews are useful to propose a series of avenues for reflection and action that could be useful for the implementation of a knowledge brokering unit like the KMTU.

**Table 1:** Six mechanisms aimed at increasing the use of research

• .Interactions between researchers and decision-makers must be considered a priority to raise awareness and build agreement on research priorities. • Use strategies like social marketing and awareness-raising campaigns to develop more positive attitudes towards research within the Ministry. • Reach a consensus on the issues of interest to the Ministry by using participatory and collaborative processes and interventions proven effective. • Improve access and communicate research results in an appropriate format, in a timely manner and using a variety of means adapted to users’ context. • Train researchers, to be able to communicate clearly to a non-research audience, and users, to be able to access and understand research results. • Change structures that influence decision-making processes to promote evidence use by policy-makers.	

### Building awareness

The first mechanism consists of strategies to make users aware of the usefulness of research and to change their opinions about it. Strategies to improve decision makers’ attitudes towards research in general are a “vital next step” [24, 25]. There is solid evidence on the effectiveness of strategies such as social marketing and awareness-raising campaigns. Such strategies could be explored and adopted by the KMTU team to develop more positive attitudes towards research within the Ministry.

### Building agreement

The second mechanism focuses on developing a common understanding of the questions of interest that research should address. Given the limitations of my mandate, I was unable to access a “research action plan” that could have informed us on the fit between research questions explored by Burkinabè researchers and the Ministry’s information needs. However, according to several respondents, studies carried out by research centres under the Ministry’s charge did not always address its priorities. Certainly, “independent” research is necessary, and not all studies should proceed to KT. However, recent study demonstrated the importance of local evidence [26] and of participatory and collaborative processes (Delphi groups, interactive forum, etc.) [27] to better target the issues of interest to the Ministry.

### Improving access and communication

The third mechanism consists of strategies to improve access and communicate research results, such as creating virtual libraries for retrieving relevant information, using social media and other online media, and putting results into appropriate formats (policy briefs, research summaries, infographics, etc.). These strategies are effective when undertaken in a timely manner [28] and when the communication strategy employs a variety of means adapted to users’ context (theatre forums, videos, deliberative workshops, tailored and targeted messaging, etc.) [27, 29]. As we saw, at the Ministry of Health,^1^ a registry of available studies will first have to be created, work that has begun in the Department of Archives and Documentation. At the heart of this strategy will be the KMTU’s role in communicating relevant results.

### Facilitating interaction

The synthesis presents limited evidence regarding the effectiveness of interactions between researchers and decision-makers to promote research use. Even if further data are needed, recent studies show that it remains highly likely that these mechanisms can influence use [27, 28]. As mentioned above, several of our recent studies in West Africa, and more particularly in Burkina Faso, have focused on evaluating strategies, such as knowledge brokering, to foster researcher–user interaction. However, as analysis of our respondents’ statements clearly shows, interactions between researchers and decision-makers must be considered a priority for the latter, and the hierarchy, particularly the Minister and his SG, will have an essential role to play in ensuring the active participation of decision-makers who are in a position to take action. While further studies are needed to demonstrate its effectiveness, the establishment of the KMTU, which is designed as a knowledge brokerage unit, is most promising. This initiative is likely to become a model for other countries in Africa and elsewhere. A major evaluation project should be planned to accompany this unit’s implementation and measure its effectiveness in promoting research use in the Ministry.

### Building skills

Mechanisms for training and development of skills among researchers, to be able to communicate clearly to a non-research audience, and among users, to be able to access and understand research results, are both effective and necessary. While the robustness of these results should encourage the development of more training for researchers and knowledge users [27, 29, 30], it is still necessary to assess their impacts on knowledge acquisition and skill development. However, these trainings alone will not lead to sustainable change if other efforts are not made to develop a real culture open to research within the Ministry.

### Changing decision-making structures and processes

Finally, the potential for success of Minister Méda’s initiative is strongly supported by the synthesis of the Alliance for Useful Evidence [8, 11]. Their results show, in fact, that the sixth mechanism for promoting evidence use by policy-makers involves changing structures that influence decision-making processes. When such changes are combined with better access to evidence and to user training activities, as is envisioned with the KMTU, they are effective in supporting evidence-based decision-making.

## Conclusions

This study aimed at understanding the decision-makers’ point of view on the use of research. The results discussed in the article allowed us to propose six avenues for reflection and action—based on Langer’s et al. work [11] to increase the potential impact of the knowledge transfer unit recently created within the Ministry of Health of Burkina Faso. This innovative initiative promises to be impactful for the implementation of evidence-based practices if obstacles identified in this study and policy-makers’ preferences and needs are taken into account. The avenues for reflection and action proposed in this article will certainly be useful to guide the implementation of other initiatives of this type.

## Data Availability

The datasets generated and analysed during the current study are not publicly available due to confidentiality constraints but are available from the corresponding author upon reasonable request.

## Abbreviations

KT: Knowledge transfer
KMTU: Knowledge management and transfer unit

## Footnotes

1 Several years ago, a registry was created as part of the EvipNet project, but this registry did not contain any keywords for navigating through it to find relevant information.
